# Exploring the link between metabolic syndrome risk and physical fitness in children with obesity: a cross-sectional study

**DOI:** 10.1007/s00431-025-06339-7

**Published:** 2025-07-24

**Authors:** Matteo Vandoni, Alessandro Gatti, Vittoria Carnevale Pellino, Luca Marin, Caterina Cavallo, Virginia Rossi, Giulia Lascialfari, Gianvincenzo Zuccotti, Valeria Calcaterra

**Affiliations:** 1 Laboratory of Adapted Motor Activity (LAMA), Department of Public Health, Experimental Medicine and Forensic Science, University of Pavia, 27100 Pavia, Italy; 2 Department of Public Health, Experimental and Forensic Medicine, National PhD Programme in One Health Approaches to Infectious Diseases and Life Science Research, University of Pavia, 27100 Pavia, Italy; 3 Department of Rehabilitation, Healthcare Institute “Città Di Pavia”, 27100 Pavia, Italy; 4 Department of Sport, LUNEX University of Applied Sciences, 50, Avenue du Parc Des Sports, 4671 Differdange, Luxembourg; 5 Pediatric Department, Vittore Buzzi” Children’s Hospital, 20154 Milan, Italy; 6 Department of Biomedical and Clinical Science, University of Milano, 20157 Milan, Italy; 7 Pediatric and Adolescent Unit, Department of Internal Medicine, University of Pavia, 27100 Pavia, Italy; 8 Laboratory for Rehabilitation and Orthopedic Surgery (LAROS), Department of Clinical Surgical, Diagnostic and Pediatric Sciences, University of Pavia, Pavia, 27100 Italy; 9 Department of Physiotherapy, Faculty of Medicine, University of Ostrava, Ostrava, 70103 Czech Republic

**Keywords:** Metabolic syndrome, Physical fitness, Children, Adolescents, Obesity

## Abstract

Given the growing relevance of physical fitness for cardiometabolic health in children with obesity and the rising prevalence of MetS, this study aimed to explore the relationship between physical fitness and MetS. Specifically, the goal was to assess the relationship between physical fitness and the severity of MetS, as measured by the MetS risk score, and to evaluate the importance of each dimension as a potential indicator of metabolic health in the pediatric population. We consecutively recruited a total of 62 children and adolescents (aged 7–17 years) with obesity (BMI z-score > 2SD). For all the patients, we evaluated cardiorespiratory fitness, muscular strength, and speed-agility. We computed the MetS risk score, a continuous risk score based on BMI z-score, HDL-C, systolic blood pressure, triglycerides, and fasting glucose, and MetS was defined as having a z-score > 0.75. Cardiorespiratory fitness and speed-agility were not significantly associated with MetS. Lower limb muscular strength, measured by the standing broad jump, was inversely associated with the odds of having MetS (OR: 0.47; 0.22–0.88; 95% CI), with a 1SD (24 cm) increase in SBJ corresponding to a 53% reduction in risk. Additionally, cardiorespiratory fitness was not related to any cardiometabolic outcomes (*p* > 0.05), whereas muscular strength showed an inverse association with the MetS risk score (*β*_2_ =  − 0.266, *p* = 0.043), BMI z-score (*β*_2_ =  − 0.443, *p* = 0.001), SBP (*β*_2_ =  − 0.304, *p* = 0.020), and waist-to-height ratio (*β*_2_ =  − 0.328, *p* = 0.012). *Conclusion*: We demonstrated a link between MetS and physical fitness in children with obesity, with muscular strength showing a stronger association compared to cardiorespiratory fitness and speed-agility. The findings support the role of muscle integrity in metabolic health, emphasizing the importance of educating individuals from an early age about daily PA to promote lifelong healthy habits.

**Table:** 

**What is Known:** • *Physical fitness is associated with improved cardiometabolic outcomes in children and adolescents with obesity.* • *Muscular strength, as one domain of physical fitness, has been linked to metabolic health, but findings are inconsistent and often context-dependent.*
**What is New:** • *This study shows that muscular strength, specifically lower limb strength measured by standing broad jump, is inversely associated with metabolic syndrome severity in children with obesity.* • *Cardiorespiratory fitness and speed-agility did not show significant associations in our sample, possibly due to limited variability or generally low levels; nonetheless, muscular strength emerged as a noteworthy fitness domain related to metabolic risk in this population. *

**Supplementary Information:**

The online version contains supplementary material available at 10.1007/s00431-025-06339-7.

## Introduction

Over the past 40 years, childhood obesity rates have surged, doubling among children aged 2–4 and increasing eightfold in those aged 5–19 worldwide [1]. Currently, approximately 18.5% of children and adolescents between the ages of 2 and 19 are affected, totaling around 13.7 million individuals [1].

Childhood obesity is associated with numerous metabolic, inflammatory, and systemic complications [2]. The excessive accumulation of adipose tissue promotes the secretion of adipokines and fosters chronic low-grade inflammation, contributing to insulin resistance, dyslipidemia, and hypertension [3]. Research indicates that nearly 75% of children with obesity remain with obesity into adulthood, significantly increasing their risk of developing metabolic and cardiovascular disorders [4–6].

The global rise in childhood obesity is driven by a complex interplay of genetic, epigenetic, and environmental factors. Key contributors include dietary imbalances and a decline in physical activity (PA) opportunities due to urbanization and the growing prevalence of digital device use during leisure time [7]. As reported, PA plays a fundamental role in maintaining a healthy lifestyle, particularly in childhood, and serves as a powerful preventive tool against obesity and its associated comorbidities [8, 9]. The World Health Organization (WHO) emphasizes the importance of promoting physical activity and an active lifestyle as a primary strategy for preventing health conditions [7, 10]. Regular exercise has been shown to enhance cardiovascular fitness and regulate inflammatory processes across all populations [11].

Indeed, PA and exercise are widely recognized for their ability to improve muscle mass and maintain metabolic function. Specifically, a direct measure of muscle functionality can be obtained by analyzing the child’s muscular strength, a domain of physical fitness (PF). PF involves various domains, including health-related and skills-related components [12], and refers to the ability to perform PA and movements efficiently and effectively. Among PF’s health-related components, muscular strength and cardiorespiratory fitness play a central role for health in children. Both cardiorespiratory fitness and muscular strength are associated with cardiovascular health and lipid metabolism [13, 14]. Aerobic and resistance exercise, specifically, stimulate the production of anti-inflammatory myokines like interleukin-6 (IL-6), which protects against systemic inflammation [15]. On the other hand, skill-related PF instead refers to a wide range of domains (i.e., speed-agility) implied in sports or activities and related to motor competence [16]. Although this categorization appears to divide PF into distinct domains, in reality, skill-related factors influence health-related performance and vice versa [17, 18].

Moreover, PA enhances insulin sensitivity, improves lipid metabolism, and reduces the risk of obesity, thereby preventing metabolic syndrome (MetS) [19].

Although there is consensus on the distinctive features of MetS, no international diagnostic criteria currently exist for the pediatric population [20]. Recently, Gurka et al. [21] proposed a MetS risk score as a highly sensitive and specific tool for detecting risk markers of MetS, which was also found to be strongly associated with the incidence of type 2 diabetes [22].

Given the relevance of physical fitness for cardiometabolic health in children with obesity and the increasing rates of MetS, understanding if there is a relation between these two outcomes could help practitioners develop targeted interventions for this population. For these reasons, the study aimed to assess a link between physical fitness and the degree of severity of the metabolic syndrome computed according to the MetS risk score, to evaluate the importance of muscular fitness as a marker of metabolic health in the pediatric population. Promoting PA and physical fitness among children and adolescents should be a key public health objective, benefiting both individual well-being and overall community health.

## Methods

### Study design

This study is a cross-sectional analysis from the “Fight against Pediatric Obesity: from a predictive tool for type 2 Diabetes and Cardiovascular diseases risk to healthy educational programs (PODiaCar)” study. The study is registered and approved by the ethical committee (Ethics committee Lombardia 1, protocol number: CET 202–2023). Each child and their parents were provided with a detailed explanation of the study’s purpose and objectives before giving consent. Verbal consent was then obtained from the children, while their parents or legal guardian provided written informed consent. The study followed the STrengthening the Reporting of OBservational studies in Epidemiology (STROBE) for reporting cross-sectional results (Table S1).

### Participants

We consecutively recruited a total of 62 children and adolescents with obesity (girls 25/62, 40.3%), referred to the pediatric outpatient clinic of Buzzi Children’s Hospital of Milan (Italy) by their general practitioner or primary care pediatrician between March 2024 and February 2025. They were considered eligible if they fulfilled the following criteria: aged between 6 and 17 years, a BMI z-score higher than 2 SD, being able to perform physical activity, and not being involved in other studies. Exclusion criteria included secondary obesity, lack of Italian language comprehension, chronic cardiovascular or respiratory diseases, orthopedic injuries that occurred in the past 6 months, any condition preventing participation in physical activity, and any medical condition that restricted the ability to engage in exercise.

### Clinical assessment

In all patients, weight, height, waist circumference (WC), pubertal stage according to Marshall and Tanner [23], BMI, and waist-to-height ratio (WHtR) were considered. During these evaluations, participants were barefoot and wore lightweight exercise clothing (shorts and a T-shirt). Body weight was measured using a balance scale (Seca 864, Seca GmbH and Co., Hamburg, Germany) with an accuracy of 0.1 kg. Height was determined with a Harpenden stadiometer (Holtain Ltd., Cross-well, Crymych, UK) with a fixed vertical backboard and adjustable headpiece [24]. WC was measured using a flexible tape measure placed horizontally at the midpoint between the lowest ribs and the iliac crest [24]. The collected data were used to calculate the BMI as body weight (kilograms) divided by height (meters squared) and BMI z-score based on the World Health Organization (WHO) Growth Chart and corresponding Equation [25]. Children were classified as having obesity if their BMI z-score exceeded 2 standard deviations, according to WHO criteria. Pubertal stages according to Tanner were classified as follows: prepubertal stage 1 = Tanner 1; middle puberty stage 2 = Tanner 2–3; and late puberty stage 3 = Tanner 4–5.

### Blood sample analysis

Fasting blood samples were collected between 8:30 and 9:00 a.m. to measure key metabolic markers: fasting blood glucose, total cholesterol, high-density lipoprotein cholesterol (HDL-C), triglycerides, and insulin levels. These samples were processed and analyzed using the Advia XPT system from Siemens Healthcare on the same day. To assess insulin resistance (IR), the Homeostatic Model Assessment for Insulin Resistance (HOMA-IR) was assessed by using the following formula: HOMA-IR = (insulin × glucose)/22.5.

To evaluate the presence and the severity of the MetS, we computed the MetS risk score as proposed by Gurka et al. [21] and we considered having MetS if the MetS risk score was higher than 0.75, a threshold used in previous studies to indicate the presence of MetS [21, 26]. This score incorporates BMI z-score, HDL-C, systolic blood pressure, triglycerides (log-transformed), and fasting glucose, computing a continuous metric of metabolic risk severity. The formula to assess the MetS risk score is presented below:

- Boys
- MetS risk score =  − 4.931 + 0.2804 * BMI z-score − 0.0257 * HDL-C + 0.0189 * Systolic blood pressure + 0.6240 * log(triglycerides) + 0.0140 * fasting glucose
- Girls
- MetS risk score =  − 4.3757 + 0.4849 * BMI z-score − 0.0176 * HDL-C + 0.0257 * Systolic blood pressure + 0.3172 * log(triglycerides) + 0.0083 * fasting glucose

### Physical fitness

All physical fitness tests were conducted in the afternoon between approximately 3:00 p.m. and 4:00 p.m. Participants were advised to arrive at the hospital completely rested. All the physical fitness tests were performed in the same day with a complete rest of 5 min between every test.

### 6-min walking test

Given the clinical setting and characteristics of our pediatric population with obesity, cardiorespiratory fitness was assessed using the 6-min walk test (6MWT), a validated submaximal test conducted following the standardized guidelines of the American Thoracic Society (ATS) [27]. Children were meticulously instructed on walking the greatest distance possible in 6 min on a straight flat surface without stopping and maintaining the same pace if possible. Standardized verbal encouragement was provided every 2 min during the test, including information on how much time remained (e.g., “You’re doing well, four minutes left!”), in line with ATS guidelines [27].

### Standing broad jump

Lower limb explosive strength was assessed using a standing broad jump (SBJ) test [28, 29]. In this test, each child started from a standing position and jumped as far as possible. To execute the jump, the children were instructed to bend their knees, extend their arms forward parallel to the ground, and then swing their arms back and forth while pushing off forcefully to achieve maximum distance. They were instructed to land with their feet together and maintain their balance. Each child performed the jump twice with a 5-min rest between the two trials, and the greatest distance was recorded in centimeters.

## × 10-m shuttle run

To assess speed and agility, the 4 × 10-m shuttle run test was conducted [29, 30]. Two parallel lines were marked 10 m apart on the ground. Participants ran back and forth as quickly as possible, ensuring both feet crossed each line each time, covering a total distance of 40 m. Before the test, evaluators provided a detailed explanation and demonstration. Time was recorded using a manual stopwatch (stopwatch W073, SEIKO, Tokyo, Japan) and stopped when the participant crossed the finish line with both feet.

### Statistical analysis

The sample size of this study was calculated using a one-tailed correlation point biserial test, considering an alpha error of 0.05, a power of 80%, and a correlation coefficient of 0.31 (following the results found by Bailey et al. [31]). To achieve the desired sample size, we had to include at least 60 children with obesity. Only children with no missing data were included in the analysis.

To assess the relationship between MetS and physical fitness levels, we computed adjusted odds ratios (ORs) using a logistic regression model. In this model, MetS served as the dependent variable, while physical fitness domains were the independent variables. Age was included as a covariate to control for potential confounding effects. Sensitivity analyses were also conducted separately by sex to explore potential differences in the associations between physical fitness and metabolic outcomes. Results of these subgroup analyses are provided in Supplementary Tables S3 and S4. This approach enabled us to evaluate the strength and direction of the association between each physical fitness domain and MetS odds while considering age differences. An OR was considered significant if its 95% confidence interval (CI) did not include 1.

To further examine the association between physical fitness domains and MetS and cardiometabolic outcomes, we conducted linear regression first performed adjusting only by age (model 1) and then with age and sex as covariates (model 2). We reported both unstandardized (*b*) and standardized (*β*) beta coefficients. Standardized coefficients were computed using the lm.beta package in R, which scales each variable to have a mean of 0 and a standard deviation of 1 prior to analysis. To further explore the relationship between MetS and PF, we performed linear regression models adjusting by age, sex, and obesity-associated outcomes and reported them in the supplementary materials (Table S5).

All analyses were conducted using R software, version 4.4 (R Foundation for Statistical Computing); the “zscorer” package was used to compute the BMIz-score, the “lm.beta” package to compute the beta coefficients of the regression, and the “oddsratio” package to assess the OR.

## Results

The descriptive characteristics of the total sample, divided by sex, are presented in Table 1. To provide further information on the sample size, we have provided the descriptive characteristics of the children with and without MetS in Table S2.

**Table 1:** Descriptive characteristics of the sample divided by sex

	Girls (*n* = 25)	Boys (*n* = 37)	Overall (*n* = 62)
Age (years)	11.5 (10.4, 12.6)	11.3 (10.7, 12.0)	11.4 (10.8, 12.0)
Height (cm)	147.9 (143.1, 152.7)	152.0 (148.4, 155.6)	150.3 (147.4, 153.2)
Weight (kg)	66.5 (59.0, 74.0)	67.6 (61.9, 73.3)	67.2 (62.7, 71.7)
BMI z-score	2.95 (2.71, 3.18)	3.17 (2.94, 3.40)	3.08 (2.91, 3.25)
Waist circumference (cm)	84.9 (80.8, 89.0)	92.0 (88.3, 95.8)	89.2 (86.3, 92.1)
WtHr	0.58 (0.55, 0.60)	0.61 (0.59, 0.62)	0.59 (0.58, 0.61)
Pubertal stage			
Stage 1	8 (32.0%)	7 (18.9%)	15 (24.2%)
Stage 2	11 (44.0%)	23 (62.1%)	34 (54.8%)
Stage 3	6 (24.0%)	7 (18.9%)	13 (21.0%)
Systolic blood pressure (mmHg)	107 (103, 111)	109 (105, 112)	109 (105, 110)
Diastolic blood pressure(mmHg)	67 (63, 71)	68 (65, 71)	68 (65, 70)
Fasting glucose (mg/dL)	87 (84, 90)	90 (88, 92)	89 (87, 91)
Fasting insulin (mg/dL)	24.8 (16.3, 33.4)	21.5 (12.9, 30.2)	22.9 (16.7, 29.0)
HOMA-IR	5.5 (3.5, 7.4)	4.9 (2.7, 7.1)	5.1 (3.6, 6.6)
Metabolic syndrome (*n* (%))	19 (76.0%)	21 (56.8%)	40 (64.5%)
Total cholesterol (mg/dL)	149 (139, 159)	155 (145, 165)	153 (146, 160)
HDL (mg/dL)	48 (44, 53)	44 (41, 47)	46 (43, 48)
Tryglicerides (mg/dL)	92 (77, 107)	103 (85, 120)	98 (86, 110)
MetS risk score	1.08 (0.86, 1.31)	0.95 (0.76, 1.14)	1.01 (0.86, 1.15)
VAI	3.61 (2.70, 4.52)	3.19 (2.53, 3.85)	3.35 (2.82, 3.89)
6MWT (m)	473 (450, 496)	473 (450, 496)	473 (457, 489)
4 × 10 shuttle run (s)	16.09 (15.50, 16.67)	15.50 (14.85, 16.15)	15.74 (15.28, 16.19)
SBJ (cm)	95 (87, 104)	107 (100, 113)	102 (97, 107)

Data were expressed as mean (95% confidence interval; CI) unless otherwise stated. *BMI* body mass index, *WtHr* waist-to-height ratio, *HOMA-IR* Homeostatic Model Assessment for Insulin Resistance, *HDL* high-density lipoprotein, *MetS* metabolic syndrome, *VAI* visceral adiposity index, *6MWT* 6-min walking test, *SBJ* standing broad jump

**Fig. 1:**
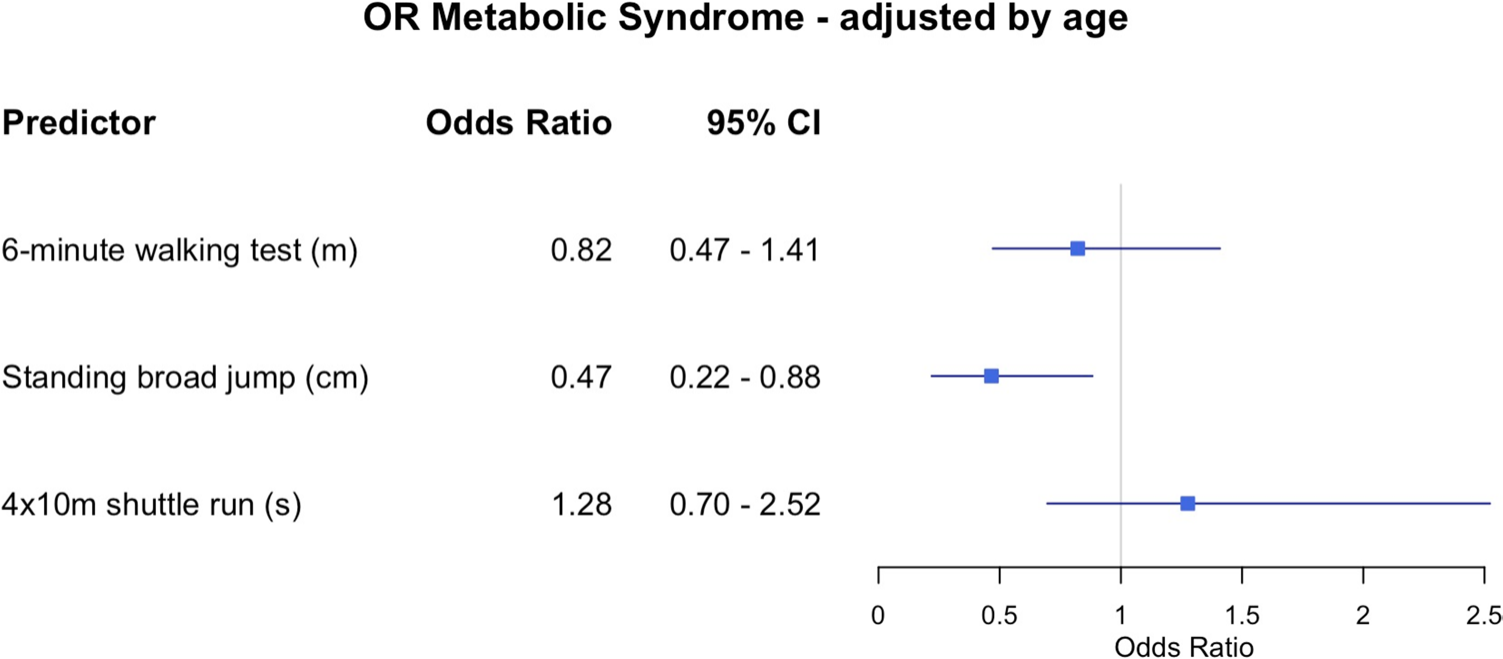
Forest plot of adjusted odds ratios (ORs) and 95% confidence intervals (CIs) for predictors of metabolic syndrome in children with obesity. Each row displays a specific predictor, with the square dot representing the adjusted for age odds ratio and the horizontal line extending from the dot indicating the 95% confidence interval. The plot includes a vertical reference line at an OR of 1.0, representing no effect. Predictors with confidence intervals that do not cross this line suggest a statistically significant association with metabolic syndrome. The numerical values of the odds ratio values and their CI are placed next to each predictor

Figure 1 shows how the different PF domains affect the odds of having MetS for children with obesity, adjusting all for age. Both cardiorespiratory fitness (6MWT) and speed-agility (4 × 10 m) were not significantly related to the odds of having MetS (6MWT OR: 0.82; 0.47–1.41; 95% CI; 4 × 10 m OR: 1.28; 0.70–2.52; 95% CI). Lower limb muscular strength (SBJ) was associated inversely with the odds of having MetS (OR: 0.47; 0.22–0.88; 95% CI), with a 1 SD (24 cm) increment in the SBJ corresponding to a 53% reduction in the odds, Fig. 1. In Figs. S1 and S2, the ORs are presented for boys and girls separately.

The associations between cardiorespiratory fitness, muscular strength, and speed-agility with cardiometabolic outcomes in children with obesity are reported in Table 2. Cardiorespiratory fitness was not related to any of the cardiometabolic outcomes (*p*-value > 0.05). Muscular strength was inversely associated with the MetS risk score (*β* (model 2) =  − 0.266, *p*-value = 0.043), BMI z-score (*β* (model 2) =  − 0.443, *p*-value = 0.001; BMC), SBP (*β* (model 2) =  − 0.304, *p*-value = 0.020), and WtHr (*β* (model 2) =  − 0.328, *p*-value = 0.012), while SBJ had a positive relation with fasting glucose (*β* (model 2) = 0.319, *p*-value = 0.018).

**Table 2:** Association between physical fitness (aerobic, muscular strength, and speed-agility) with glucose homeostasis and cardiometabolic risk factors in children with overweight/obesity

		Model 1			Model 2		
		*b* (95% CI)	*β*	*P*	*b* (95% CI)	*β*	*P*
6-min walking test (m)	BMI z-score (std)	− 0.001 (− 0.002, 0.000)	− 0.184	0.149	− 0.001 (− 0.002, 0.001)	− 0.162	0.214
	HDL (std)	0.001 (− 0.000, 0.002)	0.189	0.141	0.001 (− 0.000, 0.002)	0.232	0.073
	SBP (std)	0.000 (− 0.001, 0.002)	0.026	0.842	− 0.000 (− 0.002, 0.001)	− 0.026	0.840
	Triglycerides (std)	0.000 (− 0.003, 0.003)	0.011	0.934	0.000 (− 0.003, 0.003)	0.001	0.997
	Fasting glucose (std)	− 0.000 (− 0.001, 0.001)	− 0.021	0.874	− 0.000 (− 0.001, 0.001)	− 0.024	0.857
	MetS risk score	− 0.002 (− 0.004, 0.001)	− 0.188	0.144	− 0.002 (− 0.004, 0.000)	− 0.247	0.051
	DBP (mmHg)	− 0.018 (− 0.057, 0.022)	− 0.116	0.376	− 0.018 (− 0.057, 0.022)	− 0.116	0.378
	Fasting insulin (mU/L)	− 0.070 (− 0.167, 0.027)	− 0.187	0.154	− 0.070 (− 0.168, 0.028)	− 0.186	0.157
	HOMA-IR	− 0.017 (− 0.041, 0.007)	− 0.185	0.157	− 0.017 (− 0.041, 0.007)	− 0.185	0.160
	VAI	− 0.004 (− 0.012, 0.004)	− 0.133	0.303	− 0.004 (− 0.013, 0.004)	− 0.132	0.308
	WtHr	− 0.000 (− 0.000, 0.000)	− 0.167	0.195	− 0.000 (− 0.000, 0.000)	− 0.173	0.173
Standing broad jump (cm)	BMI z-score (std)	− 0.008 (− 0.012, − 0.004)	− 0.443	** < 0.001**	− 0.008 (− 0.012, − 0.003)	− 0.443	**0.001**
	HDL (std)	0.002 (− 0.002, 0.005)	0.128	0.322	0.003 (− 0.001, 0.006)	0.210	0.120
	SBP (std)	− 0.004 (− 0.009, 0.001)	− 0.184	0.153	− 0.006 (− 0.011, − 0.001)	− 0.304	**0.020**
	Triglycerides (std)	0.008 (− 0.001, 0.017)	0.233	0.068	0.009 (− 0.001, 0.018)	0.241	0.077
	Fasting glucose (std)	0.004 (0.001, 0.007)	0.289	**0.023**	0.004 (0.001, 0.008)	0.319	**0.018**
	MetS risk score	− 0.004 (− 0.011, 0.003)	− 0.151	0.242	− 0.007 (− 0.014, − 0.000)	− 0.266	**0.043**
	DBP (mmHg)	− 0.033 (− 0.162, 0.096)	− 0.069	0.611	− 0.047 (− 0.183, 0.089)	− 0.098	0.495
	Fasting insulin (mU/L)	0.008 (− 0.314, 0.329)	0.006	0.963	0.033 (− 0.306, 0.372)	0.028	0.846
	HOMA-IR	0.002 (− 0.077, 0.081)	0.007	0.957	0.006 (− 0.077, 0.090)	0.022	0.879
	VAI	− 0.007 (− 0.034, 0.020)	− 0.065	0.629	− 0.004 (− 0.032, 0.025)	− 0.036	0.797
	WtHr	− 0.001 (− 0.002, − 0.000)	− 0.328	**0.012**	− 0.001 (− 0.002, − 0.001)	− 0.448	**0.001**
4 × 10-m shuttle run (s)	BMI z-score (std)	0.067 (0.019, 0.115)	0.342	**0.007**	0.065 (0.013, 0.117)	0.331	**0.016**
	HDL (std)	− 0.004 (− 0.041, 0.033)	− 0.027	0.833	− 0.017 (− 0.057, 0.023)	− 0.118	0.397
	SBP (std)	0.007 (− 0.051, 0.064)	0.030	0.819	0.036 (− 0.024, 0.097)	0.162	0.235
	Triglycerides (std)	− 0.061 (− 0.165, 0.042)	− 0.151	0.242	− 0.062 (− 0.176, 0.051)	− 0.154	0.277
	Fasting glucose (std)	− 0.027 (− 0.066, 0.012)	− 0.177	0.168	− 0.031 (− 0.074, 0.011)	− 0.205	0.148
	MetS risk score	0.013 (− 0.069, 0.096)	0.042	0.745	0.056 (− 0.030, 0.142)	0.175	0.199
	DBP (mmHg)	0.754 (− 0.770, 2.278)	0.137	0.326	0.857 (− 0.703, 2.417)	0.156	0.276
	Fasting insulin (mU/L)	− 0.579 (− 4.391, 3.234)	− 0.043	0.762	− 0.778 (− 4.686, 3.131)	− 0.057	0.692
	HOMA-IR	− 0.148 (− 1.082, 0.785)	− 0.045	0.752	− 0.183 (− 1.141, 0.775)	− 0.055	0.704
	VAI	0.144 (− 0.175, 0.463)	0.124	0.371	0.125 (− 0.201, 0.452)	0.108	0.445
	WtHr	0.004 (− 0.004, 0.012)	0.132	0.335	0.006 (− 0.003, 0.014)	0.185	0.183

Model 1 was adjusted for age only, except for the MetS risk score and its components that were unadjusted. Model 2 was adjusted for age and sex, except for the MetS risk score and its components that were adjusted only by age. *b* = beta unstandardized coefficients. *β* = beta standardized coefficients. All the components of the MetS risk score were transformed following their transformation in the index’s formula. *CI* confidence interval, *HOMA-IR* homeostasis model assessment of insulin resistance, *HDL* high-density lipoprotein, *SBP* systolic blood pressure, *DBP* diastolic blood pressure

For speed-agility, we found a positive significant association only with the BMI z-score (*β* (model 2) = 0.331, *p*-value = 0.016) (Table 2). In Tables S3 and S4, the regression models (model 1 unadjusted and model 2 adjusted by age) are presented for boys and girls separately. Table S5 presents the analysis adjusted for obesity-related outcomes. After adjusting for these outcomes, the relationship between SBJ and MetS weakened (*β* (model 4) =  − 0.219, *p*-value = 0.078).

To facilitate the visualization of the results, we graphically represented the associations with regression plots reporting the *β* and the *p*-value of model 2 in Figs. 2, 3, and 4. From Figs. S3 to S8, we presented the linear regression plots for boys and girls separately.

**Fig. 2:**
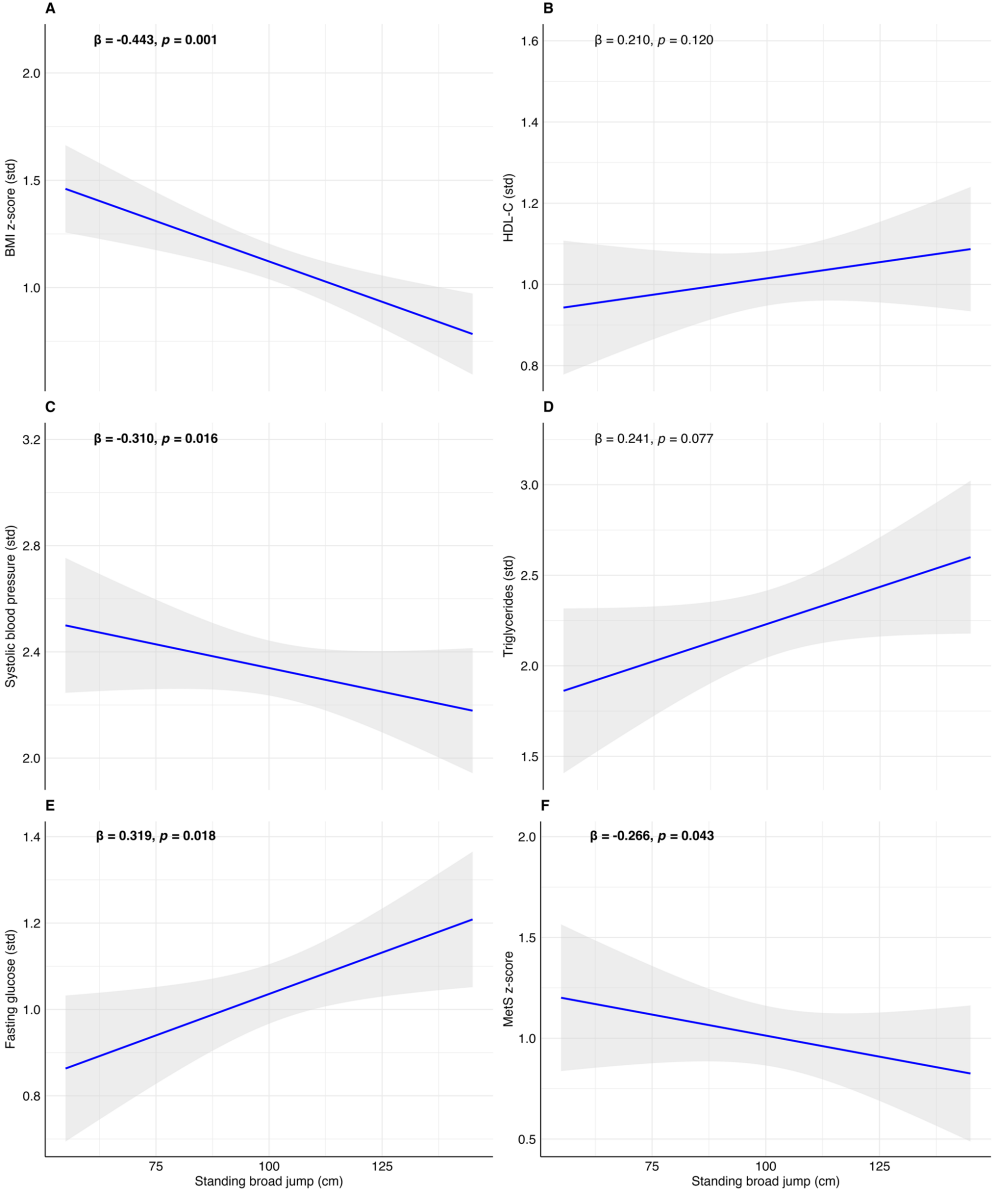
Associations between standing broad jump (cm) with BMI z-score (**A**), high-density lipoprotein (HDL-C, **B**), systolic blood pressure (**C**), triglycerides (**D**), fasting glucose (**E**), and metabolic syndrome risk score (MetS risk score, **F**) in children with obesity. Shading indicates the 95% confidence intervals of the associations. Bold beta and *p*-value indicate statistically significant associations (*p* < 0.05)

**Fig. 3:**
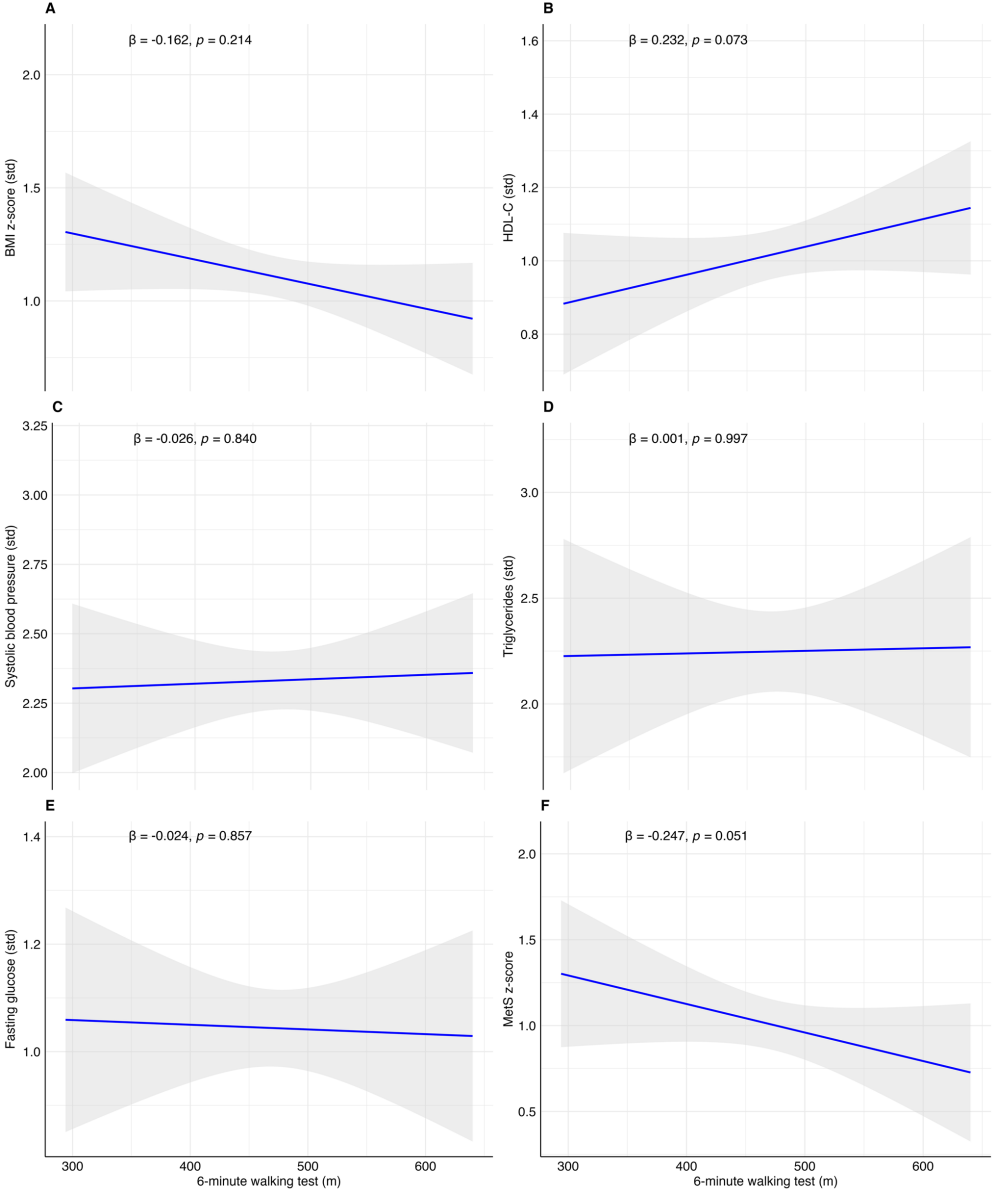
Associations between 6-min walking test (m) with BMI z-score (**A**), high-density lipoprotein (HDL-C, **B**), systolic blood pressure (**C**), triglycerides (**D**), fasting glucose (**E**), and metabolic syndrome risk score (MetS risk score, **F**) in children with obesity. Shading indicates the 95% confidence intervals of the associations. Bold beta and *p*-value indicate statistically significant associations (*p* < 0.05)

**Fig. 4:**
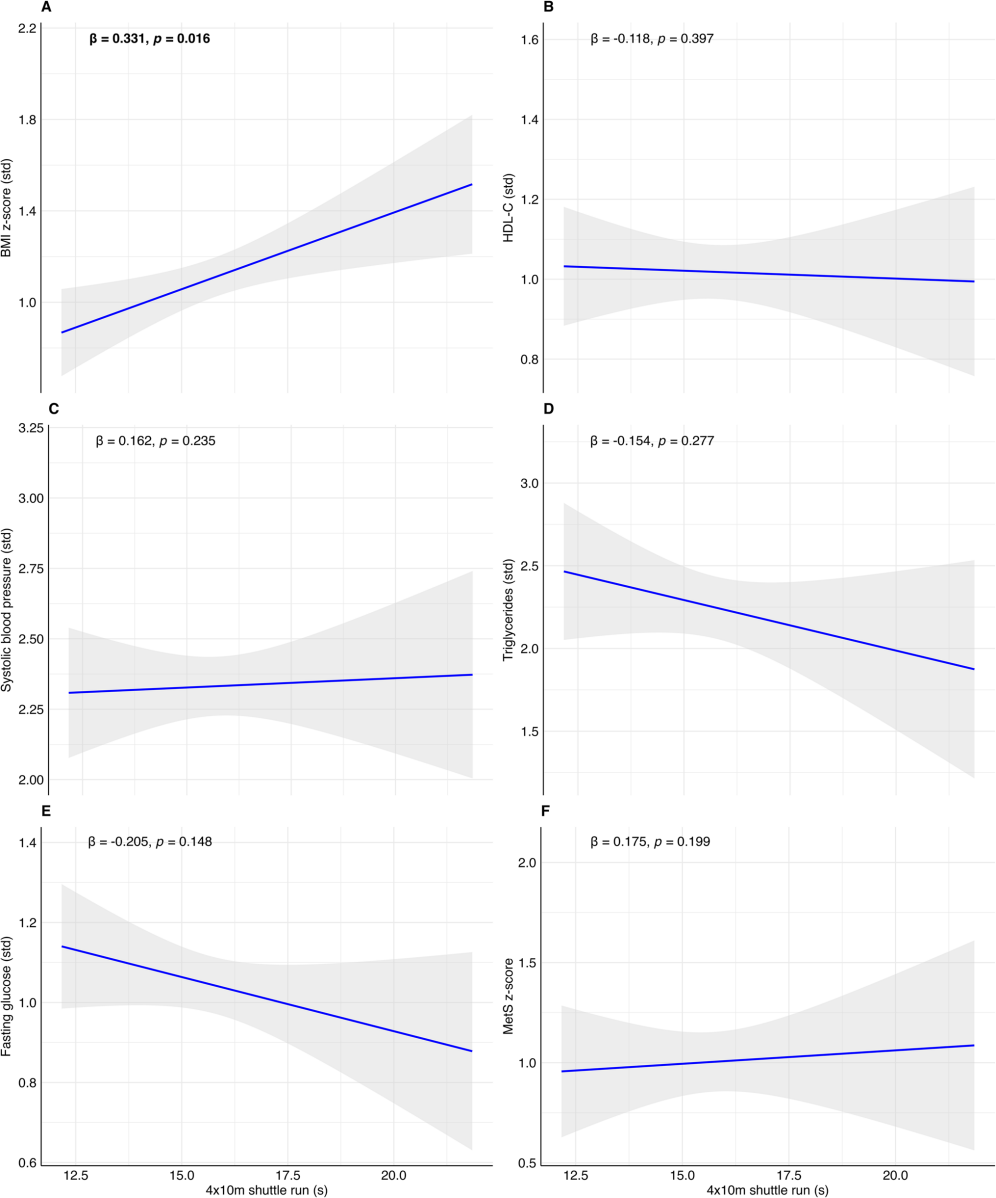
Associations between 4 × 10-m shuttle run test (s) with BMI z-score (**A**), high-density lipoprotein (HDL-C, **B**), systolic blood pressure (**C**), triglycerides (**D**), fasting glucose (**E**), and metabolic syndrome risk score (MetS risk score, **F**) in children with obesity. Shading indicates the 95% confidence intervals of the associations. Bold beta and *p*-value indicate statistically significant associations (*p* < 0.05)

## Discussion

We analyzed the link between physical fitness and the MetS risk score in children and adolescents with obesity. Our results revealed that only muscular strength was significantly associated with the odds of having MetS, while cardiorespiratory fitness and speed-agility showed no direct relationship with it. Specifically, muscular strength was significantly associated with three out of the five outcomes used to compute the MetS risk score. In contrast, cardiorespiratory fitness showed no association with any of the five MetS risk score components, whereas higher speed-agility performance was linked only to a lower BMI z-score. These findings support the hypothesis that muscle metabolic function plays a crucial role in children with obesity, emphasizing the importance of educating children about regular physical activity to maintain muscle integrity.

The prevalence of MetS among children and adolescents varies widely, ranging from 0.2 to 38.9% [32–34]. In the general pediatric population, it is estimated at 3.3%, while it rises to 11.9% in those with overweight and 29.2% in individuals with obesity [32–34]. MetS prevalence frequently mirrors obesity trends, particularly in high-income countries where obesity rates are more pronounced. Additionally, its occurrence fluctuates depending on factors such as age, sex, ethnicity, and the specific diagnostic criteria applied [20].

As reported by DeBoer et al. and Gurka et al. [21, 26], traditional criteria for diagnosing MetS have limitations, as they identify risk only when a person shows abnormalities exceeding the thresholds of the components. This approach does not consider that MetS may exist as a continuum of risk, as indicated by the increased risk in individuals with a growing number of abnormalities across different components of the syndrome. Consequently, an individual with values just below the threshold for all five components may be at greater risk than someone who exceeds the limits in three components but has normal or low levels in the other two. Gurka et al. [21] developed continuous MetS severity that is useful for monitoring changes over time within a population and identifying differences in the rate of change based on clinical status. Several studies have shown that continuous MetS scores are linked to unhealthy lifestyle factors [9] and increased cardiovascular risk [35, 36]. Additionally, these scores are associated with indicators of future disease, including abnormal insulin and glucose levels in both adults and children.

In our pediatric population, 64.5% of patients exhibited pathological levels of MetS. The muscular strength was inversely associated with the MetS risk score, BMI z-score, SBP, and WtHr, while SBJ had a positive relation with fasting glucose. The cardiorespiratory fitness was not related to any of the cardiometabolic outcomes, and for speed-agility, only a positive significant association with the BMI z-score was noted.

Regarding the muscular strength and MetS, even if explored with different methods (both MetS criteria and muscular strength assessments), several studies obtained similar results. For example, both Cohen et al. [37] and Burns et al. [38] found that upper limb strength was inversely related to metabolic risk factors, even if not analyzing the odds of MetS. The results of Castro-Pinero et al. [39] additionally strengthen our results. Indeed, they found that the SBJ was a strong predictor of all the physical fitness battery of the MetS in children across Europe. These relations between muscle could be due to the contributions of the skeletal muscle secretome, which exerts paracrine and endocrine functions that help maintain and regulate overall physiological health, including metabolic control and glucose homeostasis. Disruptions in glucose balance are central to the initiation and progression of MetS, with IR acting as a key factor connecting its components. In turn, the metabolic imbalances characteristic of MetS further impair glucose regulation, potentially advancing to type 2 diabetes [40].

Unlike previous studies that examined this relationship [39, 41], we selected absolute rather than relative standing broad jump performance. Relative measures, such as jump distance normalized to body weight, account for body composition, including lean mass, bone mass, and fat mass, making them more reflective of overall muscle fitness rather than muscle strength alone [42]. Additionally, changes in relative SBJ performance may result from variations in both muscle strength and body fat mass [42]. Therefore, absolute SBJ performance may serve as a more direct indicator of muscle strength [42] and muscular fitness [43]. Our data on the positive relation between SBJ and fasting glucose confirm the role of muscular integrity in glycemic homeostasis. The attenuation of this relationship after adjustment for obesity-related variables confirms that the influence of muscular strength on MetS is also influenced by adiposity excess [44, 45], which may limit the protective metabolic effects of muscular fitness. These results are consistent with prior research [44, 46] highlighting the mediating role of body fat in the relationship between physical fitness and cardiometabolic health. This underscores the need for interventions targeting both muscle development and fat reduction, as focusing on muscular fitness in isolation may offer limited metabolic benefit in children with high adiposity.

In our pediatric population, we found that neither cardiorespiratory fitness nor speed-agility was significantly associated with metabolic risk factors or MetS. Our results for the cardiorespiratory fitness contrast with those of previous studies. Specifically, Burns et al. [38] and Artero et al. [41] found an inverse relationship between MetS risk and cardiorespiratory fitness. This discrepancy may be due to differences in the methods used to assess cardiorespiratory fitness. While we used the 6MWT, both studies employed submaximal running tests, which require greater effort and may better differentiate children with MetS. Considering the speed-agility, even though there is less literature reporting this association [18], the results of these studies are conflicting. Sánchez-López et al. [47] found that individuals with a favorable agility classification had lower cardiometabolic risk. However, Henriksson et al. [48] proved that the relationship between MetS and speed-agility disappeared when controlling for specific obesity outcomes. One possible explanation for the discrepancy in results may be the lack of a defined MetS scoring system in children and the use of different systems to evaluate it. Therefore, future consensus and guidelines should establish a universally accepted MetS scoring system or assessment tool to reduce variability in the results of different studies and to clarify the relationship between PF and MetS in children.

We must acknowledge some limitations for our study. Firstly, our sample size compared to the sample sizes of previously published studies seems small; however, our sample size was sufficient to reach the desired statistical power. Secondly, this study followed a cross-sectional and not a longitudinal design, limiting the ability to draw causal conclusions. Instead, it allows for the identification of linear relationships between muscular strength, cardiorespiratory fitness, speed-agility, and MetS. Despite its limitations, this study has also notable strengths. Firstly, this is the first study analyzing the relationship between MetS and physical fitness in Italian children and adolescents with obesity. The study adhered to rigorous ethical standards, including the STROBE guidelines. Moreover, we included in our study a comprehensive physical fitness test battery, allowing us to evaluate three domains of health-related physical fitness and associate them with MetS in children with obesity.

In conclusion, our results showed a link between MetS and physical fitness in children with obesity, with a stronger association between muscular strength compared to cardiorespiratory fitness and speed-agility. Data support the role of muscle integrity in metabolic health, highlighting the importance of educating individuals from a young age about daily physical activity to foster lifelong healthy habits. By promoting PA and physical fitness, we can enhance and support the developmental process, leading to balanced growth, skeletal muscle health, and the prevention of diseases later in life. Developing strategies and initiatives to encourage physical activity among children and adolescents should be a primary focus for public health.

## Supplementary information

## Supplementary Information

Below is the link to the electronic supplementary material.

Supplementary file1 (DOCX 68 KB)

Supplementary file2 (DOCX 69 KB)

Supplementary file3 (DOCX 311 KB)

Supplementary file4 (DOCX 331 KB)

Supplementary file5 (DOCX 331 KB)

Supplementary file6 (DOCX 347 KB)

Supplementary file7 (DOCX 352 KB)

Supplementary file8 (DOCX 25 KB)

Supplementary file9 (DOCX 26 KB)

Supplementary file10 (DOCX 31 KB)

Supplementary file11 (DOCX 31 KB)

Supplementary file12 (DOCX 30.8 KB)

Supplementary file13 (DOCX 23.9 KB)

## Data Availability

The data that support the findings of this study are available from the corresponding author upon reasonable request.
