# Supplementary material for: Exploring the link between metabolic syndrome risk and physical fitness in children with obesity: a cross-sectional study
Source: Eur J Pediatr. 2025 Jul 24;184(8):497. doi: 10.1007/s00431-025-06339-7 (PMC12289717; doi:10.1007/s00431-025-06339-7)
Supplement: Supplementary file 2 — Supplementary file2 (DOCX 69 KB) [file 431_2025_6339_MOESM2_ESM.docx]

**
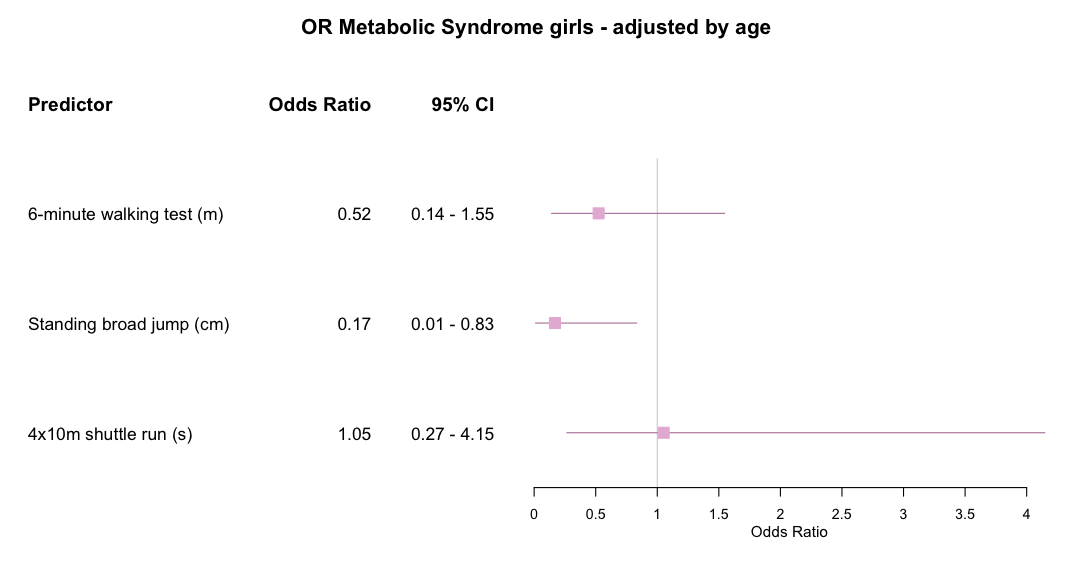
**

**Figure S2.**  Forest plot of adjusted odds ratios (ORs) and 95% confidence intervals (CIs) for predictors of metabolic syndrome in girls with obesity. Each row displays a specific predictor, with the square dot representing the adjusted for age odds ratio and the horizontal line extending from the dot indicating the 95% confidence interval. The plot includes a vertical reference line at an OR of 1.0, representing no effect. Predictors with confidence intervals that do not cross this line suggest a statistically significant association with metabolic syndrome. The numerical values of the odds ratio values and their CI are placed next to each predictor.
