# Supplementary material for: Exploring the link between metabolic syndrome risk and physical fitness in children with obesity: a cross-sectional study
Source: Eur J Pediatr. 2025 Jul 24;184(8):497. doi: 10.1007/s00431-025-06339-7 (PMC12289717; doi:10.1007/s00431-025-06339-7)
Supplement: Supplementary file 5 — Supplementary file5 (DOCX 331 KB) [file 431_2025_6339_MOESM5_ESM.docx]

**
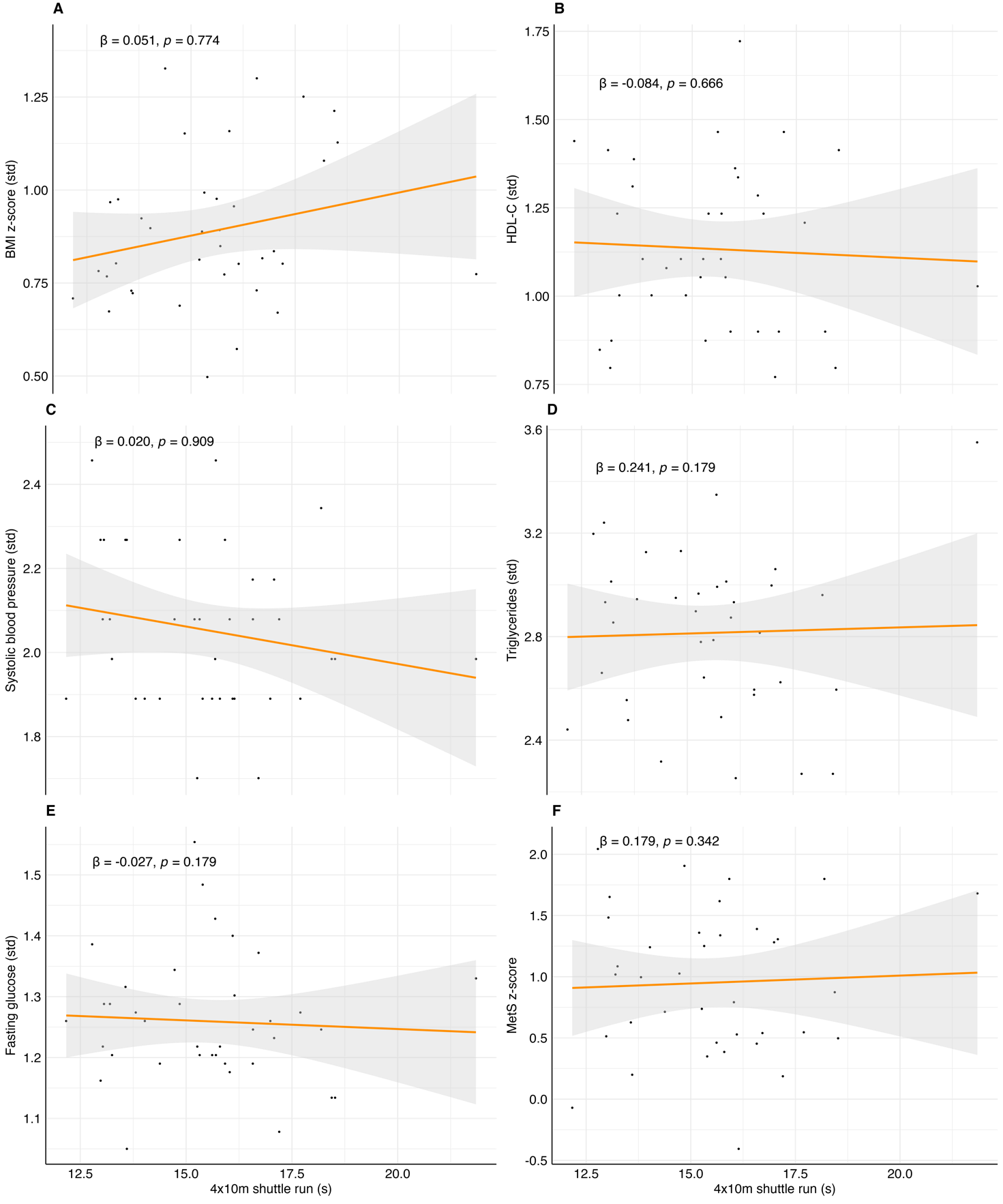
**

**Figure S5.** Associations between 4x10m shuttle run (s) with BMI z-score (panel A), High density lipoprotein (HDL-C, panel B), systolic blood pressure (panel C), triglycerides (panel D), fasting glucose (panel E) and metabolic syndrome z-score (MetS z-score, panel F) in boys with obesity. Shading indicates the 95% confidence intervals of the associations. Bold beta and p-value indicate statistically significant associations (*p* < 0.05).
