# Supplementary material for: Exploring the link between metabolic syndrome risk and physical fitness in children with obesity: a cross-sectional study
Source: Eur J Pediatr. 2025 Jul 24;184(8):497. doi: 10.1007/s00431-025-06339-7 (PMC12289717; doi:10.1007/s00431-025-06339-7)
Supplement: Supplementary file 8 — Supplementary file8 (DOCX 25 KB) [file 431_2025_6339_MOESM8_ESM.docx]

**
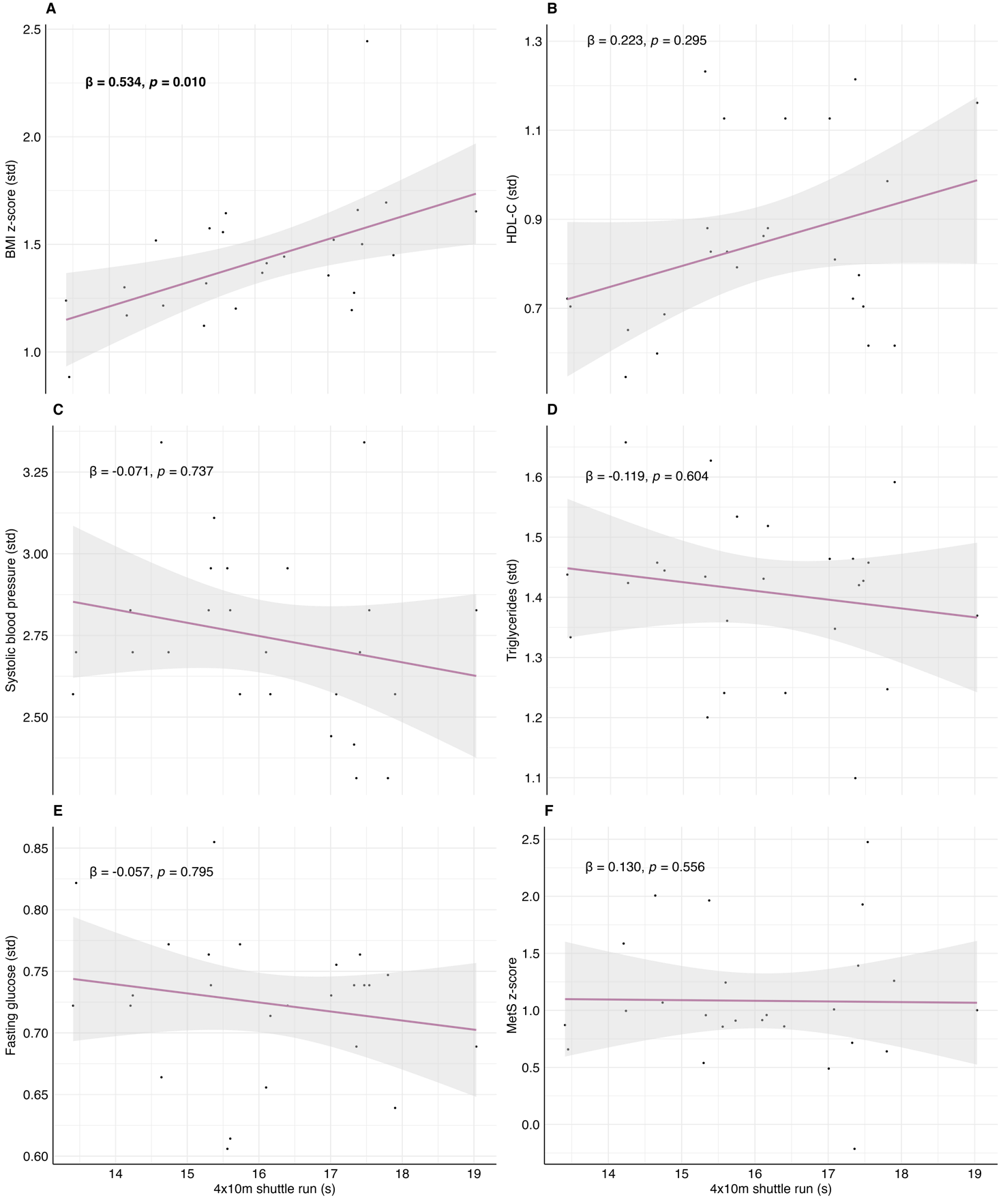
**

**Figure S8.** Associations between 6-minute walking test (m) with BMI z-score (panel A), High density lipoprotein (HDL-C, panel B), systolic blood pressure (panel C), triglycerides (panel D), fasting glucose (panel E) and metabolic syndrome z-score (MetS z-score, panel F) in girls with obesity. Shading indicates the 95% confidence intervals of the associations. Bold beta and p-value indicate statistically significant associations (*p* < 0.05).
