# Supplementary material for: Exploring the link between metabolic syndrome risk and physical fitness in children with obesity: a cross-sectional study
Source: Eur J Pediatr. 2025 Jul 24;184(8):497. doi: 10.1007/s00431-025-06339-7 (PMC12289717; doi:10.1007/s00431-025-06339-7)
Supplement: Supplementary file 10 — Supplementary file10 (DOCX 31 KB) [file 431_2025_6339_MOESM10_ESM.docx]

**Table S2.** Descriptive characteristics of the sample divided by MetS.

| **All** | **Without MetS**  **(n=25)** | **With MetS**  **(n=37)** |
| --- | --- | --- |
| Girls (n (%)) | 6 (27.3%) | 19 (47.5%) |
| Boys (n (%)) | 16 (72.7%) | 21 (52.5%) |
| Age (years) | 10.8 (10.0, 11.5) | 11.8 (10.9, 12.6) |
| Height (cm) | 147.9 (143.6, 152.2) | 151.7 (147.9, 155.5) |
| Weight (kg) | 59.2 (54.2, 64.2) | 71.6 (65.5, 77.6) |
| BMI z-score | 2.89 (2.58, 3.21) | 3.18 (2.99, 3.37) |
| Waist circumference (cm) | 84.4 (80.8, 87.9) | 92.0 (88.1, 95.8) |
| WtHr | 0.57 (0.55, 0.59) | 0.61 (0.59, 0.63) |
| Systolic blood pressure (mmHg) | 102 (98, 107) | 111 (108, 114) |
| Diastolic blood pressure(mmHg) | 63 (60, 66) | 70 (67, 73) |
| Fasting glucose (mg/dL) | 89 (86, 92) | 89 (86, 91) |
| Fasting Insulin (mg/dL) | 14.6 (11.7, 17.5) | 27.4 (18.3, 36.6) |
| HOMA-IR | 3.2 (2.5, 3.9) | 6.2 (4.0, 8.4) |
| Total cholesterol (mg/dL) | 153 (141, 164) | 153 (144, 162) |
| HDL (mg/dL) | 53 (49, 57) | 42 (39, 45) |
| Triglycerides (mg/dL) | 69 (59, 79) | 115 (99, 130) |
| MetS z-score | 0.41 (0.29, 0.54) | 1.33 (1.20, 1.46) |
| VAI | 1.85 (1.46, 2.24) | 4.20 (3.53, 4.88) |
| 6MWT (m) | 477 (448, 507) | 471 (451, 491) |
| 4x10 shuttle run (s) | 15.7 (15.0, 16.5) | 15.7 (15.2, 16.3) |
| SBJ (cm) | 108 (100, 115) | 99 (92, 106) |

Data were expressed as mean (95% confidence interval; CI) unless otherwise stated. BMI, body mass index; WtHr, waist to height ratio; HOMA-IR, Homeostatic Model Assessment for Insulin Resistance; HDL, high density lipoprotein; MetS, metabolic syndrome; VAI, visceral adiposity index; 6MWT, 6-minute walking test; SBJ, standing broad jump.
