# Supplementary material for: Exploring the link between metabolic syndrome risk and physical fitness in children with obesity: a cross-sectional study
Source: Eur J Pediatr. 2025 Jul 24;184(8):497. doi: 10.1007/s00431-025-06339-7 (PMC12289717; doi:10.1007/s00431-025-06339-7)
Supplement: Supplementary file 11 — Supplementary file11 (DOCX 31 KB) [file 431_2025_6339_MOESM11_ESM.docx]

**Table S3.** Association between physical fitness (aerobic, muscular strength and speed-agility) with glucose homeostasis and cardiometabolic risk factors in boys with overweight/obesity.

|  |  | Model 1 | | | Model 2 | | |
| --- | --- | --- | --- | --- | --- | --- | --- |
|  |  | b (95% CI) | β | P | b (95% CI) | β | P |
| 6-minute walking test (m) | BMI z-score (std) | -0.001 (-0.002, 0.000) | -0.287 | 0.085 | -0.000 (-0.001, 0.001) | -0.130 | 0.455 |
|  | HDL (std) | 0.001 (-0.001, 0.002) | 0.157 | 0.355 | 0.001 (-0.001, 0.002) | 0.215 | 0.259 |
|  | SBP (std) | 0.000 (-0.000, 0.001) | 0.160 | 0.343 | -0.000 (-0.001, 0.001) | -0.042 | 0.805 |
|  | Triglycerides (std) | 0.000 (-0.001, 0.002) | 0.084 | 0.621 | -0.000 (-0.002, 0.001) | -0.085 | 0.638 |
|  | Fasting glucose (std) | -0.000 (-0.001, 0.000) | -0.067 | 0.694 | -0.000 (-0.001, 0.000) | -0.122 | 0.522 |
|  | MetS z-score | -0.001 (-0.003, 0.002) | -0.076 | 0.656 | -0.002 (-0.005, 0.001) | -0.208 | 0.262 |
|  | DBP (mmHg) | -0.018 (-0.069, 0.033) | -0.120 | 0.481 | -0.034 (-0.091, 0.022) | -0.229 | 0.223 |
|  | Fasting insulin (mU/L) | -0.049 (-0.177, 0.079) | -0.129 | 0.445 | -0.061 (-0.205, 0.083) | -0.161 | 0.399 |
|  | HOMA-IR | -0.013 (-0.046, 0.019) | -0.141 | 0.406 | -0.017 (-0.053, 0.020) | -0.174 | 0.361 |
|  | VAI | 0.002 (-0.008, 0.012) | 0.070 | 0.680 | -0.003 (-0.013, 0.008) | -0.101 | 0.573 |
|  | WtHr | -0.000 (-0.000, 0.000) | -0.068 | 0.689 | -0.000 (-0.000, 0.000) | -0.231 | 0.206 |
|  |  |  |  |  |  |  |  |
| Standing broad jump (cm) | BMI z-score (std) | -0.003 (-0.006, 0.001) | -0.276 | 0.099 | -0.001 (-0.005, 0.002) | -0.136 | 0.420 |
|  | HDL (std) | 0.001 (-0.003, 0.005) | 0.078 | 0.646 | 0.001 (-0.003, 0.006) | 0.109 | 0.559 |
|  | SBP (std) | 0.001 (-0.002, 0.004) | 0.100 | 0.558 | -0.001 (-0.004, 0.002) | -0.083 | 0.619 |
|  | Tryglicerides (std) | -0.002 (-0.008, 0.003) | -0.141 | 0.406 | -0.005 (-0.010, 0.000) | -0.321 | 0.060 |
|  | Fasting glucose (std) | 0.000 (-0.002, 0.002) | 0.002 | 0.992 | -0.000 (-0.002, 0.002) | -0.031 | 0.869 |
|  | MetS z-score | -0.005 (-0.015, 0.005) | -0.167 | 0.324 | -0.009 (-0.020, 0.002) | -0.290 | 0.105 |
|  | DBP (mmHg) | -0.104 (-0.288, 0.080) | -0.191 | 0.257 | -0.159 (-0.355, 0.037) | -0.291 | 0.108 |
|  | Fasting insulin (mU/L) | -0.225 (-0.688, 0.238) | -0.165 | 0.330 | -0.265 (-0.772, 0.243) | -0.194 | 0.296 |
|  | HOMA-IR | -0.055 (-0.172, 0.063) | -0.157 | 0.352 | -0.064 (-0.193, 0.065) | -0.185 | 0.320 |
|  | VAI | -0.007 (-0.043, 0.029) | -0.063 | 0.710 | -0.024 (-0.060, 0.012) | -0.230 | 0.184 |
|  | WtHr | -0.001 (-0.002, 0.000) | -0.229 | 0.174 | -0.001 (-0.002, -0.000) | -0.389 | **0.025** |
|  |  |  |  |  |  |  |  |
| 4x10m shuttle run (s) | BMI z-score (std) | 0.023 (-0.010, 0.057) | 0.231 | 0.168 | 0.005 (-0.031, 0.041) | 0.051 | 0.774 |
|  | HDL (std) | -0.006 (-0.045, 0.034) | -0.048 | 0.777 | -0.010 (-0.055, 0.036) | -0.084 | 0.666 |
|  | SBP (std) | -0.018 (-0.050, 0.014) | -0.189 | 0.262 | 0.002 (-0.031, 0.035) | 0.020 | 0.909 |
|  | Tryglicerides (std) | 0.005 (-0.049, 0.058) | 0.030 | 0.858 | 0.038 (-0.018, 0.093) | 0.241 | 0.179 |
|  | Fasting glucose (std) | -0.003 (-0.021, 0.015) | -0.055 | 0.748 | -0.001 (-0.022, 0.019) | -0.027 | 0.890 |
|  | MetS z-score | 0.013 (-0.088, 0.114) | 0.044 | 0.797 | 0.053 (-0.059, 0.164) | 0.179 | 0.342 |
|  | DBP (mmHg) | 1.278 (-0.491, 3.048) | 0.241 | 0.151 | 2.084 (0.157, 4.012) | 0.392 | **0.035** |
|  | Fasting insulin (mU/L) | -0.331 (-4.894, 4.231) | -0.025 | 0.884 | -0.401 (-5.615, 4.813) | -0.030 | 0.877 |
|  | HOMA-IR | -0.089 (-1.245, 1.067) | -0.027 | 0.876 | -0.112 (-1.433, 1.209) | -0.033 | 0.864 |
|  | VAI | 0.097 (-0.252, 0.445) | 0.095 | 0.578 | 0.329 (-0.028, 0.686) | 0.322 | 0.070 |
|  | WtHr | -0.002 (-0.011, 0.007) | -0.083 | 0.626 | 0.001 (-0.009, 0.011) | 0.053 | 0.776 |

Model 1 was unadjusted. Model 2 was adjusted for age. b = beta unstandardized coefficients. β = beta standardized coefficients. All the components of the MetS z-score were transformed following their transformation in the index’s formula. CI, confidence interval; HOMA-IR, homeostasis model assessment of insulin resistance; HDL, high-density lipoprotein; SBP, systolic blood pressure; DBP, diastolic blood pressure.
