# Supplementary material for: Exploring the link between metabolic syndrome risk and physical fitness in children with obesity: a cross-sectional study
Source: Eur J Pediatr. 2025 Jul 24;184(8):497. doi: 10.1007/s00431-025-06339-7 (PMC12289717; doi:10.1007/s00431-025-06339-7)
Supplement: Supplementary file 12 — Supplementary file12 (DOCX 30.8 KB) [file 431_2025_6339_MOESM12_ESM.docx]

**Table S4.** Association between physical fitness (aerobic, muscular strength and speed-agility) with glucose homeostasis and cardiometabolic risk factors in girls with overweight/obesity.

|  |  | Model 1 | | | Model 2 | | |
| --- | --- | --- | --- | --- | --- | --- | --- |
|  |  | b (95% CI) | β | P | b (95% CI) | β | P |
| 6-minute walking test (m) | BMI z-score (std) | -0.004 (-0.008, 0.000) | -0.359 | 0.078 | -0.004 (-0.008, 0.000) | -0.387 | 0.059 |
|  | HDL (std) | 0.001 (-0.000, 0.003) | 0.365 | 0.073 | 0.001 (-0.000, 0.003) | 0.323 | 0.097 |
|  | SBP (std) | -0.000 (-0.002, 0.002) | -0.099 | 0.639 | -0.000 (-0.002, 0.002) | -0.048 | 0.809 |
|  | Triglycerides (std) | -0.000 (-0.001, 0.001) | -0.162 | 0.438 | -0.000 (-0.001, 0.001) | -0.145 | 0.497 |
|  | Fasting glucose (std) | -0.000 (-0.000, 0.000) | -0.041 | 0.846 | 0.000 (-0.000, 0.000) | 0.004 | 0.985 |
|  | MetS z-score | -0.004 (-0.008, -0.000) | -0.405 | **0.044** | -0.004 (-0.007, 0.000) | -0.371 | 0.060 |
|  | DBP (mmHg) | 0.002 (-0.066, 0.070) | 0.013 | 0.949 | 0.007 (-0.061, 0.075) | 0.043 | 0.837 |
|  | Fasting insulin (mU/L) | -0.078 (-0.235, 0.080) | -0.208 | 0.319 | -0.063 (-0.218, 0.091) | -0.170 | 0.405 |
|  | HOMA-IR | -0.015 (-0.051, 0.020) | -0.184 | 0.379 | -0.012 (-0.047, 0.023) | -0.143 | 0.481 |
|  | VAI | -0.013 (-0.029, 0.003) | -0.333 | 0.112 | -0.012 (-0.028, 0.005) | -0.311 | 0.147 |
|  | WtHr | -0.000 (-0.001, 0.000) | -0.239 | 0.260 | -0.000 (-0.001, 0.000) | -0.215 | 0.322 |
|  |  |  |  |  |  |  |  |
| Standing broad jump (cm) | BMI z-score (std) | -0.006 (-0.011, -0.001) | -0.472 | **0.017** | -0.006 (-0.012, -0.001) | -0.463 | **0.029** |
|  | HDL (std) | -0.002 (-0.006, 0.002) | -0.171 | 0.412 | -0.000 (-0.005, 0.004) | -0.052 | 0.805 |
|  | SBP (std) | 0.001 (-0.005, 0.006) | 0.065 | 0.759 | -0.001 (-0.006, 0.004) | -0.079 | 0.702 |
|  | Tryglicerides (std) | 0.001 (-0.002, 0.004) | 0.165 | 0.431 | 0.001 (-0.002, 0.004) | 0.126 | 0.574 |
|  | Fasting glucose (std) | 0.001 (-0.000, 0.002) | 0.383 | 0.059 | 0.001 (-0.000, 0.002) | 0.296 | 0.153 |
|  | MetS z-score | -0.002 (-0.013, 0.009) | -0.070 | 0.741 | -0.005 (-0.017, 0.006) | -0.195 | 0.362 |
|  | DBP (mmHg) | 0.107 (-0.070, 0.284) | 0.253 | 0.223 | 0.083 (-0.106, 0.273) | 0.197 | 0.370 |
|  | Fasting insulin (mU/L) | 0.468 (0.082, 0.854) | 0.463 | **0.020** | 0.403 (-0.006, 0.812) | 0.399 | 0.053 |
|  | HOMA-IR | 0.110 (0.024, 0.196) | 0.483 | **0.014** | 0.094 (0.003, 0.184) | 0.412 | **0.043** |
|  | VAI | 0.022 (-0.024, 0.068) | 0.210 | 0.325 | 0.018 (-0.030, 0.067) | 0.173 | 0.441 |
|  | WtHr | -0.001 (-0.002, -0.000) | -0.435 | **0.033** | -0.001 (-0.003, -0.000) | -0.524 | **0.012** |
|  |  |  |  |  |  |  |  |
| 4x10m shuttle run (s) | BMI z-score (std) | 0.104 (0.033, 0.175) | 0.534 | **0.006** | 0.107 (0.028, 0.186) | 0.548 | **0.010** |
|  | HDL (std) | 0.048 (-0.009, 0.104) | 0.339 | 0.098 | 0.031 (-0.029, 0.092) | 0.223 | 0.295 |
|  | SBP (std) | -0.040 (-0.117, 0.036) | -0.222 | 0.285 | -0.013 (-0.092, 0.066) | -0.071 | 0.737 |
|  | Tryglicerides (std) | -0.015 (-0.052, 0.023) | -0.164 | 0.435 | -0.011 (-0.053, 0.031) | -0.119 | 0.604 |
|  | Fasting glucose (std) | -0.007 (-0.024, 0.009) | -0.188 | 0.367 | -0.002 (-0.020, 0.015) | -0.057 | 0.795 |
|  | MetS z-score | -0.006 (-0.171, 0.160) | -0.015 | 0.944 | 0.050 (-0.123, 0.222) | 0.130 | 0.556 |
|  | DBP (mmHg) | -2.408 (-4.863, 0.047) | -0.390 | 0.054 | -2.168 (-4.885, 0.549) | -0.351 | 0.112 |
|  | Fasting insulin (mU/L) | -4.381 (-10.446, 1.684) | -0.297 | 0.149 | -2.954 (-9.501, 3.592) | -0.201 | 0.359 |
|  | HOMA-IR | -1.016 (-2.378, 0.347) | -0.306 | 0.137 | -0.661 (-2.121, 0.800) | -0.199 | 0.358 |
|  | VAI | -0.358 (-1.005, 0.289) | -0.238 | 0.263 | -0.297 (-1.010, 0.417) | -0.197 | 0.397 |
|  | WtHr | 0.013 (-0.003, 0.030) | 0.340 | 0.104 | 0.019 (0.002, 0.035) | 0.476 | **0.032** |

Model 1 was unadjusted. Model 2 was adjusted for age. b = beta unstandardized coefficients. β = beta standardized coefficients. All the components of the MetS z-score were transformed following their transformation in the index’s formula. CI, confidence interval; HOMA-IR, homeostasis model assessment of insulin resistance; HDL, high-density lipoprotein; SBP, systolic blood pressure; DBP, diastolic blood pressure.
