# Supplementary material for: Exploring the link between metabolic syndrome risk and physical fitness in children with obesity: a cross-sectional study
Source: Eur J Pediatr. 2025 Jul 24;184(8):497. doi: 10.1007/s00431-025-06339-7 (PMC12289717; doi:10.1007/s00431-025-06339-7)
Supplement: Supplementary file 13 — Supplementary file13 (DOCX 23.9 KB) [file 431_2025_6339_MOESM13_ESM.docx]

**Table S5.** Association between physical fitness (aerobic, muscular strength and speed-agility) with glucose homeostasis and cardiometabolic risk factors in children with overweight/obesity.

|  |  | Model 3 | | | Model 4 | | |
| --- | --- | --- | --- | --- | --- | --- | --- |
|  |  | b (95% CI) | β | P | b (95% CI) | β | P |
| 6-minute walking test (m) | BMI z-score (std) | -0.000 (-0.002, 0.001) | -0.052 | 0.663 | -0.001 (-0.002, 0.000) | -0.182 | 0.166 |
|  | HDL (std) | 0.001 (-0.000, 0.002) | 0.207 | 0.123 | 0.001 (-0.000, 0.002) | 0.233 | 0.077 |
|  | SBP (std) | -0.000 (-0.002, 0.001) | -0.030 | 0.824 | -0.000 (-0.002, 0.001) | -0.055 | 0.662 |
|  | Triglycerides (std) | 0.000 (-0.003, 0.003) | 0.034 | 0.808 | 0.000 (-0.002, 0.003) | 0.038 | 0.751 |
|  | Fasting glucose (std) | -0.000 (-0.001, 0.001) | -0.006 | 0.967 | 0.000 (-0.001, 0.001) | 0.005 | 0.969 |
|  | MetS risk score | -0.001 (-0.003, 0.001) | -0.131 | 0.263 | -0.002 (-0.004, 0.000) | -0.226 | 0.057 |
|  | DBP (mmHg) | -0.016 (-0.058, 0.025) | -0.106 | 0.439 | -0.017 (-0.057, 0.023) | -0.114 | 0.394 |
|  | Fasting insulin (mU/L) | -0.074 (-0.176, 0.029) | -0.196 | 0.156 | -0.065 (-0.161, 0.032) | -0.175 | 0.185 |
|  | HOMA-IR | -0.018 (-0.043, 0.007) | -0.199 | 0.149 | -0.016 (-0.040, 0.008) | -0.177 | 0.181 |
|  | VAI | -0.005 (-0.013, 0.004) | -0.145 | 0.283 | -0.003 (-0.011, 0.005) | -0.106 | 0.394 |
|  | WtHr | -0.000 (-0.000, 0.000) | -0.003 | 0.978 | -0.000 (-0.000, 0.000) | -0.102 | 0.129 |
|  |  |  |  |  |  |  |  |
| Standing broad jump (cm) | BMI z-score (std) | -0.006 (-0.010, -0.002) | -0.344 | **0.004** | -0.008 (-0.012, -0.003) | -0.450 | **0.001** |
|  | HDL (std) | 0.002 (-0.001, 0.006) | 0.186 | 0.178 | 0.003 (-0.001, 0.006) | 0.214 | 0.122 |
|  | SBP (std) | -0.006 (-0.011, -0.001) | -0.319 | **0.018** | -0.007 (-0.012, -0.003) | -0.378 | **0.003** |
|  | Triglycerides (std) | 0.010 (0.000, 0.019) | 0.278 | **0.046** | 0.012 (0.003, 0.020) | 0.327 | **0.008** |
|  | Fasting glucose (std) | 0.005 (0.001, 0.008) | 0.349 | **0.012** | 0.005 (0.002, 0.008) | 0.387 | **0.002** |
|  | MetS risk score | -0.005 (-0.011, 0.002) | -0.172 | 0.151 | -0.006 (-0.013, 0.001) | -0.219 | 0.078 |
|  | DBP (mmHg) | -0.041 (-0.183, 0.102) | -0.085 | 0.570 | -0.045 (-0.189, 0.099) | -0.096 | 0.532 |
|  | Fasting insulin (mU/L) | 0.042 (-0.313, 0.397) | 0.036 | 0.813 | 0.113 (-0.237, 0.464) | 0.098 | 0.520 |
|  | HOMA-IR | 0.007 (-0.080, 0.094) | 0.024 | 0.873 | 0.022 (-0.064, 0.108) | 0.078 | 0.610 |
|  | VAI | -0.004 (-0.034, 0.026) | -0.043 | 0.773 | 0.008 (-0.021, 0.036) | 0.074 | 0.603 |
|  | WtHr | -0.001 (-0.001, -0.000) | -0.278 | **0.011** | -0.001 (-0.001, -0.000) | -0.187 | **0.013** |
|  |  |  |  |  |  |  |  |
| 4x10m shuttle run (s) | BMI z-score (std) | 0.047 (-0.000, 0.095) | 0.241 | 0.052 | 0.062 (0.010, 0.114) | 0.320 | **0.021** |
|  | HDL (std) | -0.013 (-0.054, 0.027) | -0.092 | 0.518 | -0.016 (-0.057, 0.024) | -0.115 | 0.422 |
|  | SBP (std) | 0.038 (-0.024, 0.100) | 0.169 | 0.227 | 0.034 (-0.023, 0.092) | 0.157 | 0.240 |
|  | Triglycerides (std) | -0.074 (-0.190, 0.041) | -0.183 | 0.204 | -0.061 (-0.164, 0.041) | -0.153 | 0.236 |
|  | Fasting glucose (std) | -0.035 (-0.078, 0.009) | -0.226 | 0.117 | -0.031 (-0.072, 0.010) | -0.204 | 0.131 |
|  | MetS risk score | 0.027 (-0.051, 0.105) | 0.084 | 0.495 | 0.050 (-0.029, 0.130) | 0.162 | 0.208 |
|  | DBP (mmHg) | 0.812 (-0.802, 2.426) | 0.148 | 0.318 | 0.774 (-0.798, 2.347) | 0.143 | 0.328 |
|  | Fasting insulin (mU/L) | -0.879 (-4.924, 3.165) | -0.065 | 0.665 | -1.198 (-5.042, 2.646) | -0.090 | 0.535 |
|  | HOMA-IR | -0.193 (-1.185, 0.799) | -0.058 | 0.698 | -0.281 (-1.224, 0.662) | -0.087 | 0.553 |
|  | VAI | 0.135 (-0.203, 0.472) | 0.116 | 0.428 | 0.090 (-0.225, 0.405) | 0.078 | 0.569 |
|  | WtHr | 0.001 (-0.006, 0.008) | 0.038 | 0.730 | 0.003 (-0.001, 0.008) | 0.102 | 0.166 |

Model 1 was adjusted for age, sex and BMI z-score, except for the MetS severity score and its components that were adjusted by age and BMI z-score. Model 2 was adjusted for age, sex and waist circumference, except for the MetS severity score and its components that were adjusted by age and waist circumference. b = beta unstandardized coefficients. β = beta standardized coefficients. All the components of the MetS severity score were transformed following their transformation in the index’s formula. CI, confidence interval; HOMA-IR, homeostasis model assessment of insulin resistance; HDL, high-density lipoprotein; SBP, systolic blood pressure; DBP, diastolic blood pressure.
